# Origin and Dynamics of HIV-1 Subtype C Infection in India

**DOI:** 10.1371/journal.pone.0025956

**Published:** 2011-10-10

**Authors:** Chengli Shen, Jodi Craigo, Ming Ding, Yue Chen, Phalguni Gupta

**Affiliations:** 1 Department of Infectious Diseases and Microbiology Graduate School of Public Health, University of Pittsburgh, Pittsburgh, Pennsylvania, United States of America; 2 Capital Medical University Beijing Youan Hospital, Beijing, China; 3 Center for Vaccine Research, and Department of Microbiology and Molecular Genetics, University of Pittsburgh, Pittsburgh, Pennsylvania, United States of America; Queensland Institute of Medical Research, Australia

## Abstract

**Objective:**

To investigate the geographical origin and evolution dynamics of HIV-1 subtype C infection in India.

**Design:**

Ninety HIV-1 subtype C *env* gp120 subtype C sequences from India were compared with 312 *env* gp120 reference subtype C sequences from 27 different countries obtained from Los Alamos HIV database. All the HIV-1 subtype C *env* gp120 sequences from India were used for the geographical origin analysis and 61 subtype C *env* gp120 sequences with known sampling year (from 1991 to 2008) were employed to determine the origin of HIV infection in India.

**Methods:**

Phylogenetic analysis of HIV-1 *env* sequences was used to investigate the geographical origin and tMRCA of Indian HIV-1 subtype C. Evolutionary parameters including origin date and demographic growth patterns of Indian subtype C were estimated using a Bayesian coalescent-based approach under relaxed molecular clock models.

**Findings:**

The majority of the analyzed Indian and South African HIV-1 subtype C sequences formed a single monophyletic cluster. The most recent common ancestor date was calculated to be 1975.56 (95% HPD, 1968.78–1981.52). Reconstruction of the effective population size revealed three phases of epidemic growth: an initial slow growth, followed by exponential growth, and then a plateau phase approaching present time. Stabilization of the epidemic growth phase correlated with the foundation of National AIDS Control Organization in India.

**Interpretation:**

Indian subtype C originated from a single South African lineage in the middle of 1970s. The current study emphasizes not only the utility of HIV-1 sequence data for epidemiological studies but more notably highlights the effectiveness of community or government intervention strategies in controlling the trend of the epidemic.

## Introduction

The first AIDS case in India was detected in 1986 among sex workers in Chennai, Tamil Nadu [1], and since then HIV-1 infection has been reported in all of the states and union territories in India. According to the UNAIDS 2010 report, India – which has a population of about 1.1 billion, has approximately 2.4 million people living with HIV-1. This makes India one of the largest HIV-1 infected populations in the world. About 90% of people newly infected with HIV-1 in India are believed to have acquired infection during unprotected sex, but HIV-1 transmission through contaminated needles and intravenous drug use is the major mode of HIV-1 transmission in the country's north-eastern states (UNGASS country progress report 2008). Genetic analyses of HIV-1 circulating in different parts of India have shown that the predominant proportion of HIV-1 circulating in India is of subtype C origin with a small fraction made up of subtypes A and B [2], [3], [4], [5], [6], [7], [8]. A previous genetic study compared subtype C sequences from India to subtype C sequences sampled from Botswana, Burundi, South Africa, Tanzania, and Zimbabwe. Overall, HIV-1 subtype C sequences from different parts of India were more closely related to each other than to subtype C sequences from other regions [6]. These results indicate that subtype C sequences in India are distinct from subtype C sequences sampled from other countries. The fact that HIV-1 isolated from different parts of India at different times are closely related [6] suggests that the preponderance of subtype C viruses over other subtypes is most probably not due to continual introductions of HIV-1 subtype C into the country, recent immigration, or representative of a cluster of isolated individuals. Recently, studies have shown that HIV-1 subtype C or recombinant subtype C that prevailed in China, Myanmar, and Taiwan were related to India [9]. Therefore, revealing the geographic origin, date of origin, and evolutionary history of HIV-1 in this region would provide valuable insight regarding the epidemic in India.

There was a previous study related to the geographical origin of Indian HIV-1 subtype C based on only two HIV-1 subtype C sequences from Africa, and 10 other HIV-1 subtype sequences around the world [10]. With the availability of large numbers of HIV-1 *env* sequences from different regions of India and the world, it is currently possible to determine more accurately the geographical origin of HIV-1 subtype C in India. Furthermore, the time to the most recent common ancestor and the dynamics of HIV-infected population over time will provide us information about HIV-1 subtype C epidemic and prevention.

In this study using phylogenetic methodologies, we estimated the origin and history of population dynamics of HIV-1 subtype C in India. Our results showed that subtype C originated in India about ten years before the first case was discovered. Moreover, there was about ten years of exponential population growth of HIV-1 in infected patients before strict measures were taken to control the virus.

## Methods

### Ethics Statement

Since the sequences used in this study were collected from the Los Alamos Data base (a public domain information) which has no patient identifier, there is no need for consent form.

### Sequence collection and phylogenetic analyses

HIV-1subtype C sequences were downloaded from the Los Alamos National Laboratory database (http://www.hiv.lanl.gov/content/index). Sequences without geographical information were discarded from the analysis. HIV-1 subtype C sequences from India were separated and manually selected in order to maximize the length of the segment analyzed as well as the number of sequences. According to this criterion, 90 Indian HIV-1 subtype C viral sequences spanning about 1400 bp of the envelope gp120 were selected. The worldwide reference sequences were separated by country and were imported into Molecular Evolutionary Genetics Analysis software version 5.0 (MEGA 5.0) [11] individually. Representative sequences from a country were selected according to the cluster in the phylogenetic tree. In a cluster, only one sequence was selected as a representative. The final non-Indian dataset was composed of 312 sequences. All selected sequences were aligned in Clustal W, manually edited when necessary and jMODELTEST program [12], [13] was used to select the best-fit model for nucleotide substitution, resulting in the GTR+I+Γ model in the dataset. A maximum-likelihood tree was constructed under the selected model, as implemented in Fast Tree 2.0 [14]. The confidence values of tree branches were tested by using Shimodaira-Hasegawa test, which can provide sufficient accuracy information as traditional bootstrap and is very fast for large data [15]. The tree was edited and displayed using FigTree v1.3.1.

### Estimation of evolutionary rates and origin dates

A Bayesian Markov Chain Monte Carlo (MCMC) approach implemented in BEAST v1.6.1 [16] was used to estimate the HIV-1 Indian C evolution characteristics. The evolutionary parameters were estimated by using 61 sequences data in chronological time-scale (from year 1991 to 2008) from HIV-1 subtype C infected Indian patients. The estimate of nucleotide substitution model parameters, evolutionary rate (μ, nucleotide substitutions per site per year, subst/site per year) and time to the most recent common ancestor (tMRCA) were estimated with a Bayesian Skyline coalescent tree prior [17], under the GTR+I+Γ model of nucleotide substitution, and a relaxed molecular clock (uncorrelated Lognormal model). Two separate MCMC chains were run for 2×10^8^ generations for each dataset, with a 10% burn-in. BEAST output was analyzed using TRACER v1.4, with uncertainty in parameter estimates reflected by the 95% highest probability density (HPD) values. The Effective Sample Size (ESS) values for estimates were more than 100.

## Results

### Origin of HIV-1 subtype C in India

**Figure 1:**
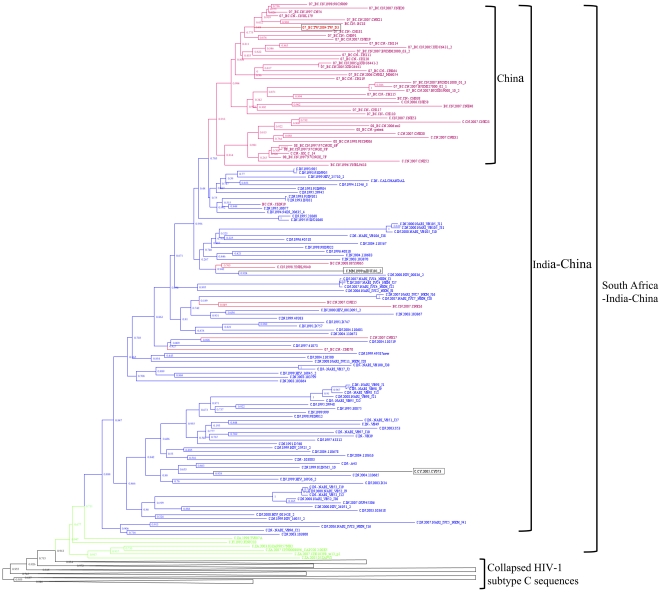
Phylogenetic analysis of env (gp120) sequences from worldwide represent samples of HIV-1subtype C. The ML tree of subtype C was inferred by using GTR+G+I model from an alignment of sequences from 28 different nations. The black triangles represent collapsed reference sequences unrelated to “South Africa-India-China” cluster. There were five Indian sequences dispersed in the collapsed cluster. SH-like support values are showed on the nodes. Brackets indicate geographic region of sampling. Line colors: Red, China; Blue, India; Green, South Africa. The names of the sequences are showed on the tips of the branches. The name of three sequences in India-China cluster are marked in black rectangles (C.MM1999mIDU101_3, 07_BC.TW2004.TW_D3 and C.CY2005.CY073), which are from Taiwan, Myanmar and Pakistan respectively.

To investigate the geographical origin of the Indian subtype C epidemic, 90 HIV-1 subtype C *env* (1400 nt) sequences from India and 312 *env* reference subtype C sequences from 27 different countries (Burundi, Brazil, Botswana, China, Belgium, Cyprus, Djibouti, Denmark, Spain, Ethiopia, Finland, Great Britain, Georgia, Israel, Kenya, Myanmar, Malawi, Nigeria, Senegal, Somalia, Thailand, Uganda, USA, Uruguay, Yemen, South Africa, and Zambia) were used to perform phylogenetic analysis. Phylogenetic analysis of the *env* (gp120) gene supported the grouping of most of the Indian and all China subtype C isolates into a single monophyletic cluster (“India-China” cluster) with a high support value of 88.8% (Figure 1). It confirmed and extended previous findings based on a very limited number of sequences that the epidemic of HIV-1 subtype C in China originated from India [18]. Moreover, three sequences, one from Myanmar and one from Taiwan were also in the “Indian-China” cluster (Figure 1), supporting a common ancestry for those isolates [18] [9]. The other sequence from Cyprus, where the patient was infected from Pakistan, was also in the India-China cluster, supporting the idea that this isolate is closely related to Indian C as reported by Kousiappa et al [19]. Six sequences were closely clustered with the “India-China” cluster with a high support value (94.7%) (Figure 1). Of the six sequences, five of them were from South Africa and one is from Finland, which originated from Africa [20]. These isolates formed a “South Africa-India-China” cluster. These results suggested that South Africa was most likely the source of introduction of HIV-1 subtype C in India. Unfortunately, there was little information available about these six patients. There were five Indian C sequences that were divergent from the “South Africa-India-China” cluster (Figure S1). Therefore, the currently accepted monophyletic structure of HIV-1 subtype C in India was questionable.

### Date of origin of HIV-1 infection in India

To explore the origination date of the HIV-1 subtype C epidemic in India, we utilized 61 HIV-1 Indian sequences of known sample date that fell within in the “India-China” cluster. A Bayesian MCMC phylogenetic analysis was conducted. Analyses were performed with Bayesian Skyline Population (BSP) models under a relaxed clock model. The mean rate of 8.31×10^−3^ nucleotide substitutions per site per year produced an average estimate of the date of origin of the Indian HIV-1 subtype C *env* sequences in the year 1975 (95% highest posterior density HPD, 1968.78–1981.52) (Table 1). The lower confidence limit of coefficient of variation was 0.373, indicating a statistically significant variability in evolution rate among lineages.

**Table 1:** Bayesian estimates of population dynamics and evolutionary parameters for HIV-1 subtype C in India.

Parameter	Estimates
**Sample size**	61
**Sample date range (year)**	1991–2008
**Demographic model**	Exponential growth(relaxed molecular clock)
**MCMC chain length**	100000000
**Mean substitution rate**	8.31×10^−3^
	(6.4×10^−3^–1.03×10^−2^)
**MRCA (year)**	32.44(26.48–39.23)
**Actual time**	1975.56
	(1959.91–1973.87)
**Coefficient of variation**	0.384
	(0.373–0.596)

95% HPD are indicated in parenthesis.

### Population dynamic history

**Figure 2:**
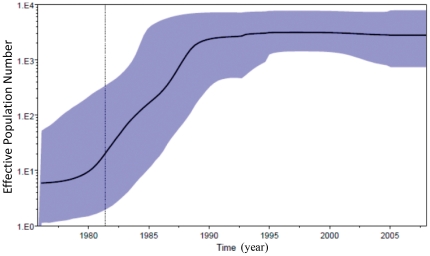
Bayesian skyline plot (BSP) of Indian and China HIV-1 subtype C. The plot begins at the median posterior Year 1975. The solid line is the traced median effective population size over a 30 year period with the 95% highest posterior density (HPD) interval. Bayesian skyline plot with relaxed molecular clock and non-parametric (BSP) estimates of demographic history were performed with BEAST version v1.7 by running two MCMC for 100,000,000 generations with sampling every 1,000th generation. The dotted vertical line is the lower 95% HPD on Indian C TMRCA.

A BSP coalescent tree prior enables the estimation of the effective population size from the sequence data of infected Indian subjects as it progresses from the origin of the epidemic through time. Phylodynamic reconstruction of the demographic history from the BSP model using *env* sequences with known sampling dates can be used to assess the population dynamics of the HIV-1 subtype C infected populace. Effective population size is the number of infections actually contributing to new infections, rather than the total number of infected individuals. The BSP analysis (Figure 2) identified three epidemic growth phases: an initial slow growth phase in 1975 to 1980 during the first five years after introduction, followed by an exponential growth phase in 1980 to 1990 and an asymptotic phase approaching the present time. The plateau since 1990 to present time cannot be unambiguously interpreted as constant growth because of little information available about changes in effective population size, but it does reflect the harmonic mean of effective population size in this period [21].

## Discussion

HIV-1 subtype C is currently considered the most prevalent international genetic form of HIV-1. It has increasingly disseminated in all continents and already accounts for half of worldwide HIV-1 infections. It predominates widely in southern Africa [22], [23], India [6], and South America [24], [25], [26]. Previously, one study using a limited number of sequences demonstrated that the introduction of HIV-1 subtype C in India was likely a single event, because all of the sequences formed a monophyletic cluster [10]. However, in that study, all of the Indian sequences analyzed were isolated within a short time frame (from 1991 to 1992) and within the same geographic region. Additionally, the study included only two HIV-1 subtype C sequences from Africa and 4 other HIV-1 subtype reference sequences including subtype A, B, D and E. The availability of more HIV-1 subtype C sequences from India and other countries provided a good opportunity to undertake a new, comprehensive analysis of the origin and evolution characteristics of HIV-1 subtype C in India.

Phylogenetic analysis showed that majority of Indian HIV-1 subtype C formed a monophyletic cluster, with close ancestral linkage to the South African isolates, forming a “South Africa-India- China” cluster. We did find five sequences that were outside of the “South Africa-India-China” cluster. These sequences were distributed in the different geographic regions. This scenario supports the notion that majority of the HIV-1 subtype C from India originated from one lineage, and some minor isolates were from separate, multiple introductions. In the “India-China” cluster, the majority of the Chinese HIV-1 subtype C and BC recombinant isolates with subtype C envelope were clustered together with some of them intermingling with Indian C sequences. The study presented here, utilizing available current sequences from the Los Alamos HIV database further confirmed Luo et al's report [18] that subtype C HIV-1 from China was closely related to Indian subtype C. The India- China cluster is likely the result of transmigration of people between China and India via Burma across the north eastern part of India. Such transmigration is also responsible for the incidence of HIV-1 infection and its recombinants among intravenous drug abusers [27], [28]. Additionally, sequences from Ethiopia, Burundi and Brazil formed one closely related cluster with a support value of 81.3% (Figure S1). The sequences from Brazil were clustered together with a support value of 99.5%, and quite related to sequences from Burundi, (supporting value 90.9%, Figure S1), similar to that reported by Fontella et al [29] and Bello et al [30].

In Asia, the highest number of HIV-1 subtype C infections were diagnosed in India where the first HIV-1 subtype C patient was diagnosed in 1986 [31]. Estimating the date of origin and predicting the past demographic characteristics of HIV infection are important for our understanding of the dynamics of the HIV-1 epidemic in India. Based on our analysis we estimate the year 1975 as the date of origin of Indian C, roughly a decade before the earliest documented Indian HIV-1 infection. The time period is similar to findings of HIV-1 subtype B infection in the USA [21].

The evolution rate has an effect on the prediction of the viral origin date. Our estimated evolution rate is 8.3×10^−3^. The substitution rate of full length *env* gp160 of HIV-1 group M estimated by Korbe et al [32] was 2.4×10^−3^, while the substitution rate for *env* gp160 of subtype B estimated by Robbins [21] was 4.73×10^−3^, and *env* gp160 of subtype C estimated by Bello et al [30] was 6.0×10^−3^. The substitution rates calculated by Leitner et al [33] for a set of V3 sequences of subtype B *env* was 6.7×10^−3^. These differences may be related to the different subtype, geographic region, gene region, the model of substitution, the homogeneity in the data set used for estimation. Because the gp120 sequences we analyzed, lack some of the more conserved regions found in the gp41 of gp160s, and the full gp120 sequences contain all five variable regions, the evolution rate estimated in our report is somewhat higher (8.3×10^−3^) when compared to those previous studies.

The demographic history of HIV-1 infection in India showed that in the first five years, from 1975 to 1980, the effective infection population grew very slowly and remained undiagnosed. In the course of the following ten years, from 1980 to 1990, the effective infection population increased exponentially. During that time period the first case was reported (year1986). At the end of 1980s, a rapid spread of HIV-1 was observed. Throughout the exponential growth period, the nation did not take measures to control spread of HIV-1 infection (http://www.avert.org/aidsindia.htm). The asymptotic growth phase after 1990s was correlated with the establishment of NACO (the National AIDS Control Organization) to organize the HIV prevention and control program in India. In 1990's, the Indian government also launched a Strategic Plan, the National AIDS Control Program (NACP) for HIV-1 prevention. This plan established the administrative and technical basis for program management and also set up State AIDS Control Societies (SACS) in 25 states and 7 union territories. These programs were able to make a number of important improvements in HIV-1 prevention such as improving blood safety. Although the total number of infections still increased after 1990, the rate decreased. Recently the number of infected patients by UNAIDS reported calculation indicates that it has begun to decrease: from 2.73 million in year 2002 to 2.40 million in year 2009 (http://www.unaids.org/globalreport/Global_report.htm).

As mentioned by Dalai et al [34] there are limitations for using Bayesian coalescent methods to estimate population demographic during the epidemic period. The presence of recent deleterious mutations from population may result an overestimation of the time to the most recent ancestor. Moreover, because of the inherent uncertainties in the phylogenetic trees, such as variable substitutions rates among viral lineages and possible difference in demographic history of viruses used in the tree, inference based on the tree should be considered along with other supporting evidence. Unlike the demographic analysis done for HIV-1 in Zimbabwe, the analysis for HIV-1 India was not restricted for any particular risk group. HIV-1 sequences were derived mainly from sexually transmitted infection and some from IV drug abusers from the north eastern corner of India.

In summary, phylogenetic analysis showed that HIV-1 subtype C from India originated from South Africa. The evolutionary reconstruction and demographic analysis of the Indian epidemic advocates that a major lineage entered the country in the middle 1970s and then transmitted in India and its border countries. The data reveal a novel perspective on the origin and evolutionary history of the subtype C epidemic in India. It further demonstrates the application of the globally sampled viral sequence data in revealing the transmission of HIV-1 across international borders. Finally, the stabilization of the growth phase of the epidemic concomitant with the organization of the HIV prevention and control program in India further emphasizes the importance of community intervention strategies in light of the ever-increasing worldwide pandemic.

## Supporting Information

Figure S1 **Phylogenetic analysis of env (gp120) sequences from worldwide represent samples of HIV-1subtype C.** The ML tree of subtype C was inferred by using GTR+G+I model from an alignment of sequences from 28 different nations. China (red), India (blue), sequences from South Africa relate to Indian isolates (green). The values on the nodes are SH-like supports value. Close related sequences from Brazil (light blue) and from Burundi (light red) are in the red rectangle box. Sequences from Ethiopia (purple) and some sequences from Burundi (light red) are in the green rectangle box.
(PDF)
