# Supplementary figures and images for: Origin and Dynamics of HIV-1 Subtype C Infection in India
Source: PLoS One. 2011 Oct 10;6(10):e25956. doi: 10.1371/journal.pone.0025956 (PMC3189977; doi:10.1371/journal.pone.0025956)

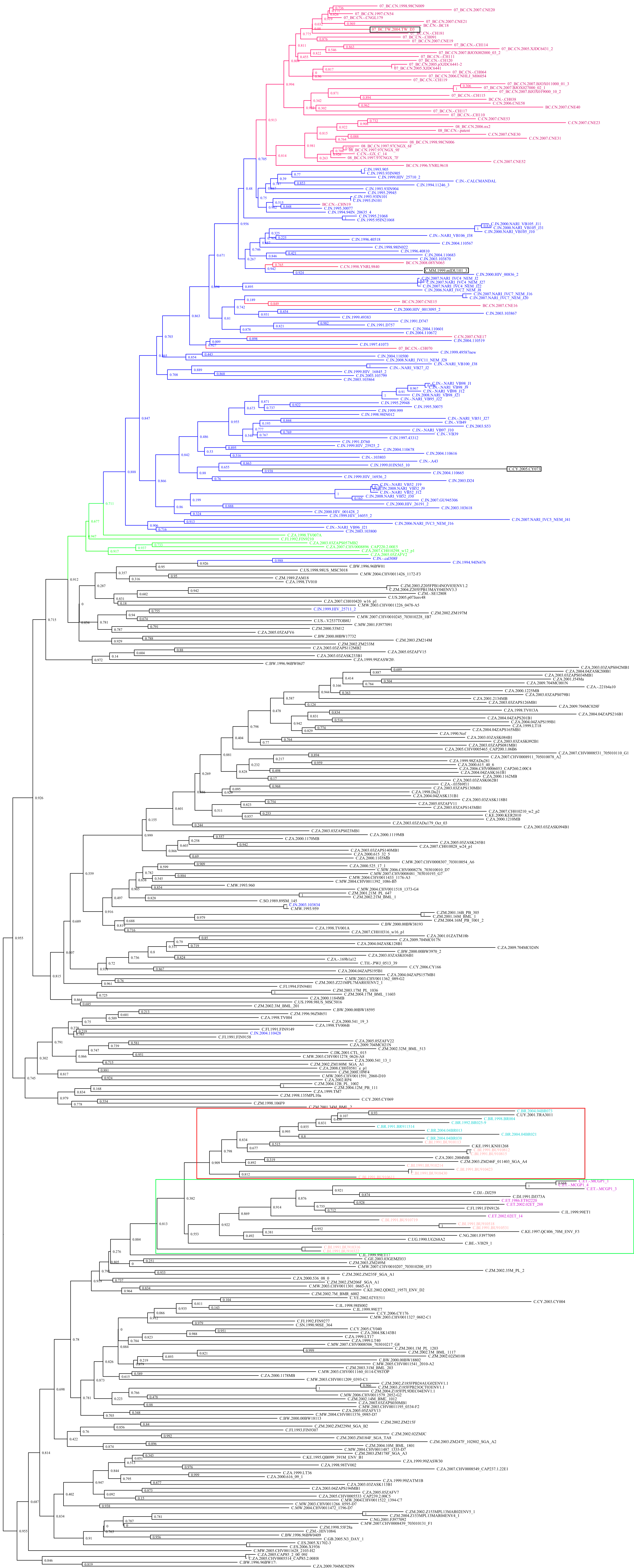

Supplement: Figure S1 — Phylogenetic analysis of env (gp120) sequences from worldwide represent samples of HIV-1subtype C. The ML tree of subtype C was inferred by using GTR+G+I model from an alignment of sequences from 28 different nations. China (red), India (blue), sequences from South Africa relate to Indian isolates (green). The values on the nodes are SH-like supports value. Close related sequences from Brazil (light blue) and from Burundi (light red) are in the red rectangle box. Sequences from Ethiopia (purple) and some sequences from Burundi (light red) are in the green rectangle box. (PDF) [file pone.0025956.s001.pdf]
